# Genome-wide identification of the fatty acid desaturases gene family in four *Aspergillus* species and their expression profile in *Aspergillus oryzae*

**DOI:** 10.1186/s13568-018-0697-x

**Published:** 2018-10-15

**Authors:** Wen Tang, Changsheng Ouyang, Lanlan Liu, Haoran Li, Chuanhui Zeng, Jie Wang, Lijun Fu, Qinqin Wu, Bin Zeng, Bin He

**Affiliations:** 1 Jiangxi Key Laboratory of Bioprocess Engineering and Co-Innovation Center for In-vitro Diagnostic Reagents and Devices of Jiangxi Province, College of Life Sciences, Jiangxi Science & Technology Normal University, Nanchang, 330013 China; 2 Nanchang University, Nanchang, 330013 China; 3 Jiangxi Provincial People’s Hospital, Nanchang, 330006 China

**Keywords:** Fatty acid desaturases, Genome-wide, Phylogenetic analysis, Expression patterns

## Abstract

Fatty acid desaturases play a key role in producing polyunsaturated fatty acids by converting single bonds to double bonds. In the present study, a total of 13, 12, 8 and 8 candidate fatty acid desaturases genes were identified in the *Aspergillus oryzae*, *Aspergillus flavus*, *Aspergillus fumigatus* and *Aspergillus nidulans* genomes through database searches, which were classified into five different subfamilies based on phylogenetic analysis. Furthermore, a comprehensive analysis was performed to characterize conserved motifs and gene structures, which could provide an intuitive comprehension to learn the relationship between structure and functions of the fatty acid desaturases genes in different *Aspergillus* species. In addition, the expression pattern of 13 fatty acid desaturases genes of *A. oryzae* was tested in different growth stages and under salt stress treatment. The results revealed that the fatty acid desaturases genes in *A. oryzae* were highly expressed in adaptive phase growth and up-regulated under salt stress treatment. This study provided a better understanding of the evolution and functions of the fatty acid desaturases gene family in the four *Aspergillus* species, and would be useful for seeking methods to improve the production of unsaturated fatty acids and enhance efforts for the genetic improvement of strains to adapt to the complex surrounding environment.

**Electronic supplementary material:**

The online version of this article (10.1186/s13568-018-0697-x) contains supplementary material, which is available to authorized users.

## Introduction

Unsaturated fatty acids, which contain one or more double bonds, are the major structural components of cell membranes. Therefore, they play significant roles in maintaining cell structure and the membrane fluidity, which are involved in development, energy metabolism and stress response (Pereira et al. 2003). Unsaturated fatty acids are synthesized by individual fatty acid desaturases via introducing double bonds into the hydrocarbon chains of fatty acids (Chi et al. 2011; Shanklin and Cahoon 1998). Fatty acid desaturases are found in almost all organisms, including plants, animals, bacteria and fungi. According to localization and cofactor requirements, fatty acid desaturases have been broadly classified into two evolutionary groups: soluble and membrane-bound desaturases. The soluble fatty acid desaturases, such as the plant Acyl-carrier-protein (ACP) desaturase family, use acyl carrier protein thioesters as substrates, and use ferredoxin oxidoreductase and ferredoxin as electron donors. The membrane-bound fatty acid desaturases, which include Δ5-, Δ6-, Δ9-, Δ12- and Δ15-desaturase in the mammals, fungi, insects, higher plants and cyanobacteria, use fatty acids esterified to complex lipid as the substrate, and use cytochrome (cyt) b5 oxidoreductase and cyt b5 as electron donors. In addition, most of fatty acid desaturases share three highly conserved histidine boxes: ʻHXXXXHʼ, ʻHXXHHʼ and ʻQXXHHʼ included in the fatty acid desaturases domain. The fatty acid desaturases domain was the essential domain of the fatty acid desaturases gene family. However, some researches revealed that the cytochrome b5 domain play a key role in the synthesis of unsaturated fatty acids as an electron donor to activate desaturase (Pereira et al. 2003). Zhang et al. reported that the cytochrome b5 is required for biosynthesis of polyunsaturated fatty acids in *Caenorhabditis elegans* (Zhang et al. 2005). Although fatty acid desaturases orthologs of different organisms share some obvious structure characteristics, the structural and functional features were distinctive among plants, animals and fungi. Most studies of fatty acid desaturases gene family focused on plants and animals. For example, Liu et al. characterized 19 genes encoding fatty acid desaturases and analyzed their expression profiles in *Gossypium raimondii* under low temperature (Liu et al. 2015). Xue et al. cloned and characterized fatty acid desaturases gene family from *Salvia hispanica* and *Perilla frutescens* (Xue et al. 2018).

Compared with unsaturated fatty acids production in animals and plants, microbes possess many advantages in the production of polyunsaturated fatty acids. For instance, unsaturated fatty acids production in microbes has a short production cycle and is unaffected by sites, climates and seasons. Besides, it is suitable to exploit new functional lipid using diverse strains and culture medium. Furthermore, lower eukaryotes contain diverse fatty acid desaturases to produce polyunsaturated fatty acids (PUFA). Therefore, in recent years, microbial fatty acid desaturases have attracted great attention of researchers. Sakuradani et al. isolated and cloned ∆6 desaturase gene from *Mortierella alpina IS*-*4*. Then the ∆6 desaturase gene was expressed in *Aspergillus oryzae* (*A. oryzae*) and the results showed that the content of gamma linolenic acid (GLA) in total fatty acids was up to 25.2% (Sakuradani et al. 1999). Sakuradani et al. improved arachidonic acid (ARA) production by generating mutants with lower desaturation activity derived from *Mortierella alpine* (Sakuradani et al. 2004). On the other hand, researches related to the function of fatty acid desaturases from fungus on stress response are also a hotspot. Cheawchanlertfa et al. revealed that the up-regulated expression of desaturase genes from *Mucor rouxii* was responded to low temperature (Cheawchanlertfa et al. 2011). The fatty acid desaturases genes in *A. oryzae* were up-regulated in response to salinity stress (He et al. 2017a). In brief, the fatty acid desaturases genes in fungi are responsible for multiple biological processes, from development and industrial production to adaption to the surrounding environment. However, systematic investigations of the fatty acid desaturases gene family at the whole-genome level was absent in fungi, especially in *Aspergillus* species, as *Aspergillus* species always encounter complex environments, artificially or non-artificially introduced (He et al. 2018).

With the price reduction of genome sequencing and the development of sequencing technology, the genome sequences were increasing available to provide opportunities for identifying important gene families at the whole-genome level. *Millerozyma farinosa* formerly known as *Pichia farinosa*, unlike *Saccharomyces cerevisiae* (containing a sole unsaturated fatty acid), contained multiple unsaturated fatty acids. It is a salt-tolerant and osmo-tolerant diploid yeast, of which the full genome sequence was completed in 2012 (Leh et al. 2012). The genome sequencing of *A. oryzae, A. flavus, A. fumigatus* and *A. nidulans* were completed earlier (Machida et al. 2005; Payne et al. 2008). On the other hand, the increasing availability of transcriptome data provides unprecedented opportunities to study the expression patterns of the members of gene family. For example, Dou et al. (2014) analyzed the expression profiles of *WRKY* gene family in different tissues of cotton through the transcriptome data.

Therefore, based on the related genomic data, we respectively identified fatty acid desaturases gene family members from two yeasts, including *S. cerevisiae* and *M. farinosa*, and four *Aspergillus* species, including *A. oryzae, A. flavus, A. fumigatus* and *A. nidulans.* In addition, a comprehensive analysis was performed to characterize conserved motifs and gene structures. Then, according to the transcriptome analysis of *A. oryzae*, the expression patterns of *A. oryzae* fatty acid desaturases gene family under salt stress and different growth periods were studied, and verified by qRT-PCR. The results of this study are propitious to comprehend the relationship between structure and functions of the fatty acid desaturases genes.

## Materials and methods

### Identification of fatty acid desaturases genes

The genomic and protein sequences of *M. farinosa CBS 7064*, *A. oryzae 304*, *A. flavus NR3357, A. fumigatus Af293*, and *A. nidulans FGSC A4* were downloaded from the National Center for Biotechnology Information (NCBI). The fatty acid desaturases protein sequences of *S. cerevisiae* (*ScFAD*) and *M. farinosa (MfFAD)* were retrieved from *Saccharomyces* Genome Database (Cherry et al. 2012). To identify all candidate fatty acid desaturases genes in *A. oryzae, A. flavus, A. fumigatus* and *A. nidulans*, *ScFAD* and *MfFAD* proteins were employed as query sequences to search genome database using BLAST program with a threshold e-value of 1e−10 (Altschul et al. 1997). Then the identity and cover region (more than 50%) were used as a filter criteria to eliminate improper fatty acid desaturases genes. Subsequently, the Pfam database was used for domain analysis to ensure that the selected sequences were non-redundant sequences to ultimately identify candidate fatty acid desaturases gene family members (Finn et al. 2014).

### Multiple sequence alignment and phylogenetic analysis

Multiple sequence alignments of fatty acid desaturases proteins in *A. oryzae, S. cerevisiae, M. farinosa, A. flavus, A. fumigatus* and *A. nidulans* were performed using Clustal X version 2.0 with the default parameters (Larkin et al. 2007). MEGA 5.0 was further applied to construct an unrooted Neighbor-Joining phylogenetic tree with pairwise deletion option and poisson correction model. Bootstrap analysis with 1000 replicates was used to examine the statistical reliability (Saitou and Nei 1987; Tamura et al. 2011). The Figtree program (v1.4.3) was used to visualize it.

### Analysis of conserved motifs and gene structures

To identify the conserved motifs of each fatty acid desaturases gene in the six species, deduced fatty acid desaturases protein sequences were subjected to MEME version 4.12.0 (http://meme-suite.org/tools/meme), with the default parameters except the number of motifs was chosen 5 (Bailey et al. 2009). The logo of motifs was produced by weblogo (http://weblogo.berkeley.edu/logo.cgi).

To illustrate exon–intron organization for each fatty acid desaturases gene, coding sequences (CDSs) and corresponding genomic sequences of fatty acid desaturases genes in the six species, downloaded from NCBI database, were compared on the Gene Structure Display Server (GSDS, http://gsds.cbi.pku.edu.cn) (Guo et al. 2007).

### Expression analysis of *AoFAD* genes in different growth stages and under salt stress treatment

The genome-wide transcriptome data of *A. oryzae* in different growth stages and salt stress treatment were obtained from NCBI SRA databases under Bioproject Accession PRJNA407002 and PRJNA383095. The raw reads that contained adapters, reads containing unknown sequences ‘N’ with a rate more than 5% and low-quality bases which were identified based on CycleQ 30 were removed. After filtering, gene expression levels were normalized using the TopHat/Cufflinks pipeline with FPKM (Fragments Per Kilobase of transcript per Million mapped reads) value (He et al. 2015). An FPKM filtering cutoff of 1.0 in at least one of the collected samples was used to determine expressed transcripts. The heatmaps for expression profiles were generated with the OmicShare Tools (http://www.omicshare.com/tools/Home/Index/index.html).

To further confirm the expression level of 13 fatty acid desaturases genes in *A. oryzae 3042* (CICC 40092), quantitative real-time RT-PCR (qRT-PCR) experiments were performed. The genome-wide transcriptome data of *A. oryzae* were obtained at three stages of development (24, 48 and 72 h) and different conditions (cultivated in potato dextrose agar medium supplied with 0, 5, 10 and 15% NaCl). Three stages of development correspond to the adaptive phase, logarithmic phase, and stationary phase. And four conditions represent control, slight stress, moderate stress and severe stress, respectively. Samples under salt stress treatment were all harvested at 48 h. Total RNA of all collected samples was extracted using PrimeScript RTreagent kit (Takara, Dalian, China) following the instructions, in which our previous studies have been performed (He et al. 2017a). The specific primers for fatty acid desaturases genes in *A. oryzae* were listed in Additional file 1: Table S1. The qRT-PCR analysis was performed on a CFX96 Real-Time PCR Detection System (Bio-Rad, CA, USA) in the BioRad CFX Connect Optics Module Real-time PCR System (Livak and Schmittgen 2001).

## Results

### Identification of fatty acid desaturases genes in the six species

The candidate fatty acid desaturases genes were identified from the *A. oryzae, A. flavus, A. fumigatus* and *A. nidulans* genome using the Blast programs with the query sequences of *S. cerevisiae* and *M. farinosa* fatty acid desaturases genes. Subsequently, the retrieved sequences were submitted to the Pfam databases to confirm the presence of conserved domains. A total of 13, 12, 8 and 8 candidate fatty acid desaturases genes were identified in the *A. oryzae, A. flavus, A. fumigatus* and *A. nidulans* genomes, respectively (Table 1). For convenience, the fatty acid desaturases genes in *A. oryzae* were named from *AoFAD1* to *AoFAD13*, these genes in *M. farinosa, A. flavus, A. fumigatus* and *A. nidulans* were named *MfFAD*, *AflFAD*, *AfuFAD* and *AnFAD* respectively. To obtain accurate sequences of fatty acid desaturases gene family, the FA_desaturase domain (PF00487) was used as a filter criteria. The results showed that the 1, 13, 12, 12, 8 and 8 fatty acid desaturases genes from *S. cerevisiae, A. oryzae, M. farinosa, A. flavus, A. fumigatus* and *A. nidulans* were all contained FA_desaturase domain, and Cyt_b5 domain was harbored in some fatty acid desaturases genes of each species. Except for the presence of conserved FA_desaturase domain and Cyt_b5 domain, Lipid_DES domain was existed in *AoFAD1*, *AflFAD1*, *AfuFAD1*, and *AnFAD1*. In addition, *AoFAD9* and *AnFAD5* respectively contained DUF953 and DUF3474 domain. The detailed information of fatty acid desaturases genes in the six species was provided in Table 1.

**Table 1:** The FAD family members in the six species

Nomenclature	Accession number in NCBI	Length of CDS	FAD group	Protein length (aa)	Domain number	Domain
AoFAD1	EIT78262.1	1266	ΙΙ-A	421	2	Lipid_DES FA_desaturase
AoFAD2	EIT82118.1	1191	Ι-B	396	2	FA_desaturase Cyt-b5
AoFAD3	EIT81402.1	1683	ΙΙ-B-2	560	2	Cyt-b5 FA_desaturase
AoFAD4	EIT77666.1	1401	ΙΙ-B-1	466	1	FA_desaturase
AoFAD5	EIT77504.1	1371	Ι-B	456	2	FA_desaturase Cyt-b5
AoFAD6	EIT77130.1	1410	Ι-B	469	2	FA_desaturase Cyt-b5
AoFAD7	EIT75420.1	1179	ΙΙ-B-1	392	1	FA_desaturase
AoFAD8	EIT74178.1	1677	ΙΙ-B-2	558	2	Cyt-b5 FA_desaturase
AoFAD9	EIT73664.1	1554	Ι-B	517	2	FA_desaturase DUF953
AoFAD10	EIT73811.1	1752	ΙΙ-B-2	583	2	Cyt-b5 FA_desaturase
AoFAD11	EIT79919.1	1032	Ι-A	343	1	FA_desaturase
AoFAD12	EIT80397.1	1059	Ι-A	352	1	FA_desaturase
AoFAD13	EIT73679.1	918	Ι-A	305	1	FA_desaturase
ScFAD	NP_011460.3	1533	Ι-B	510	2	FA_desaturase Cyt-b5
MfFAD1	XP_004200213.1	1458	Ι-B	485	2	FA_desaturase Cyt-b5
MfFAD2	XP_004199354.1	1458	Ι-B	485	2	FA_desaturase Cyt-b5
MfFAD3	XP_004205233.1	1587	Ι-B	528	2	FA_desaturase Cyt-b5
MfFAD4	XP_004204675.1	1587	Ι-B	528	2	FA_desaturase Cyt-b5
MfFAD5	XP_004195443.1	1746	ΙΙ-B-2	581	2	Cyt-b5 FA_desaturase
MfFAD6	XP_004194342.1	1743	ΙΙ-B-2	580	2	Cyt-b5 FA_desaturase
MfFAD7	XP_004197824.1	1131	Ι-A	376	1	FA_desaturase
MfFAD8	XP_004196793.1	1131	Ι-A	376	1	FA_desaturase
MfFAD9	XP_004202466.1	915	Ι-A	304	1	FA_desaturase
MfFAD10	XP_004201841.1	915	Ι-A	304	1	FA_desaturase
MfFAD11	XP_004201407.1	978	Ι-A	325	1	FA_desaturase
MfFAD12	XP_004200776.1	978	Ι-A	325	1	FA_desaturase
AflFAD1	XP_002377170.1	1266	ΙΙ-A	421	2	Lipid_DES FA_desaturase
AflFAD2	XP_002379176.1	1209	Ι-B	402	2	FA_desaturase Cyt-b5
AflFAD3	XP_002372605.1	1371	Ι-B	456	2	FA_desaturase Cyt-b5
AflFAD4	XP_002382647.1	1410	Ι-B	469	2	FA_desaturase Cyt-b5
AflFAD5	XP_002379029.1	1683	ΙΙ-B-2	560	2	Cyt-b5 FA_desaturase
AflFAD6	XP_002377775.1	1677	ΙΙ-B-2	558	2	Cyt-b5 FA_desaturase
AflFAD7	XP_002380192.1	1401	ΙΙ-B-1	466	1	FA_desaturase
AflFAD8	XP_002384911.1	1179	ΙΙ-B-1	392	1	FA_desaturase
AflFAD9	XP_002385335.1	1719	ΙΙ-B-2	572	2	Cyt-b5 FA_desaturase
AflFAD10	XP_002373264.1	1032	Ι-A	343	1	FA_desaturase
AflFAD11	XP_002378226.1	852	Ι-A	283	1	FA_desaturase
AflFAD12	XP_002385334.1	918	Ι-A	305	1	FA_desaturase
AfuFAD1	XP_752132.1	1476	ΙΙ-A	491	2	Lipid_DES FA_desaturase
AfuFAD2	XP_748918.1	1371	Ι-B	456	2	FA_desaturase Cyt-b5
AfuFAD3	XP_747146.1	1683	ΙΙ-B-2	560	2	Cyt-b5 FA_desaturase
AfuFAD4	XP_749348.1	1698	ΙΙ-B-2	565	2	Cyt-b5 FA_desaturase
AfuFAD5	XP_752623.1	1410	ΙΙ-B-1	469	1	FA_desaturase
AfuFAD6	XP_747771.1	1191	ΙΙ-B-1	396	1	FA_desaturase
AfuFAD7	XP_749168.1	1008	Ι-A	335	1	FA_desaturase
AfuFAD8	XP_747563.1	1059	Ι-A	352	1	FA_desaturase
AnFAD1	XP_662009.1	1257	ΙΙ-A	418	2	Lipid_DES FA_desaturase
AnFAD2	XP_664335.1	1368	Ι-B	455	2	FA_desaturase Cyt-b5
AnFAD3	XP_661739.1	1350	Ι-B	449	2	FA_desaturase Cyt-b5
AnFAD4	XP_662196.1	1647	ΙΙ-B-2	548	2	FA_desaturase Cyt-b5
AnFAD5	XP_658641.1	1281	ΙΙ-B-1	426	2	DUF3474 FA_desaturase
AnFAD6	XP_664808.1	1185	ΙΙ-B-1	394	1	FA_desaturase
AnFAD7	XP_664110.1	1059	Ι-A	352	1	FA_desaturase
AnFAD8	XP_661242.1	933	Ι-A	310	1	FA_desaturase

### Classification and phylogenetic analysis of the fatty acid desaturases genes

**Fig. 1:**
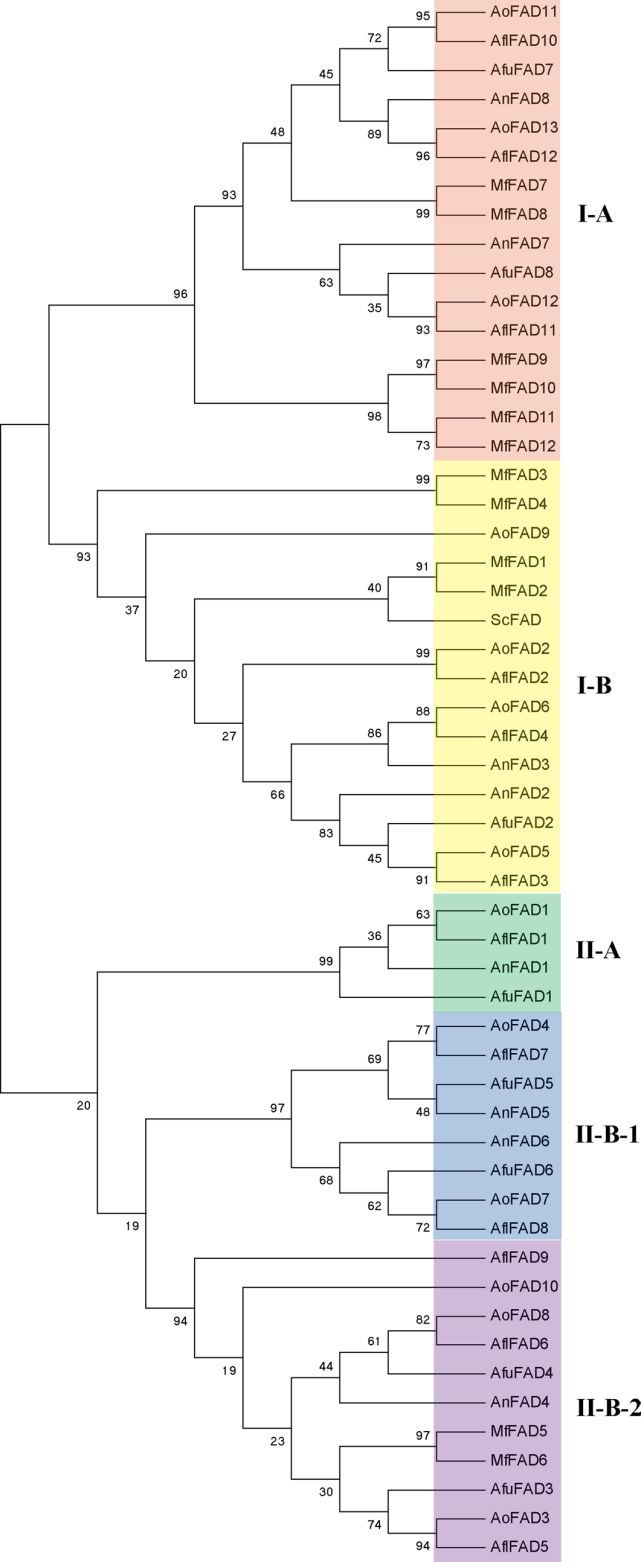
The phylogenetic tree construction of fatty acid desaturases genes. A Neighbor-Joining (NJ) phylogenetic tree of all detected fatty acid desaturases genes was constructed, using MEGA 5.0 program with bootstrap analysis (1000 replicates). Fatty acid desaturases genes in the phylogenetic tree were clustered into five distinct groups (groups I-A, I-B, II-A, II-B-1 and II-B-2)

To evaluate the phylogenetic relationships among the fatty acid desaturases gene members, all the genes from the six species were aligned separately by Neighboring-Joining method to generate an un-rooted phylogenetic tree. As shown in the phylogenetic tree (Fig. 1), the fatty acid desaturases genes in these six species were divided into two groups, which were named I and II group. According to the homology of the fatty acid desaturases genes, groups I and II were respectively further divided into I-A, I-B and II-A, II -B-1, II-B-2. Group I was composed of 31 fatty acid desaturases genes, while group II contained 23 fatty acid desaturases genes. Phylogenetic analysis of fatty acid desaturases genes in the six species revealed considerable diversification and conservation of the fatty acid desaturases gene family in fungi. In the phylogenetic tree, *ScFAD*, fatty acid desaturase in *S. cerevisiae*, was clustered with *MfFAD1* and *MfFAD2* into one branch, which belongs to group I-B. Every two fatty acid desaturases genes of *M. farinosa* shared one subbranch, which suggested that the fatty acid desaturases genes of *M. farinosa* had a strong conservation. From the phylogenetic tree, the 12 fatty acid desaturases genes from *A. flavus* were all clustered with *A. oryzae* into a subbranch, which revealed the close relationship between the *A. oryzae* and *A. flavus*, while *AoFAD9* grouped closely with *MfFAD1, 2, 3* and *4*. In addition, fatty acid desaturases genes between *A. fumigatus* and *A. nidulans* appeared to be more closely than the two other *Aspergillus*. These results can not only illustrate the relationship between the *Aspergillus* species and yeast, but also provide a potential method to distinguish *A. oryzae* and *A. flavus*.

### Conserved motifs analysis of the fatty acid desaturases genes

**Fig. 2:**
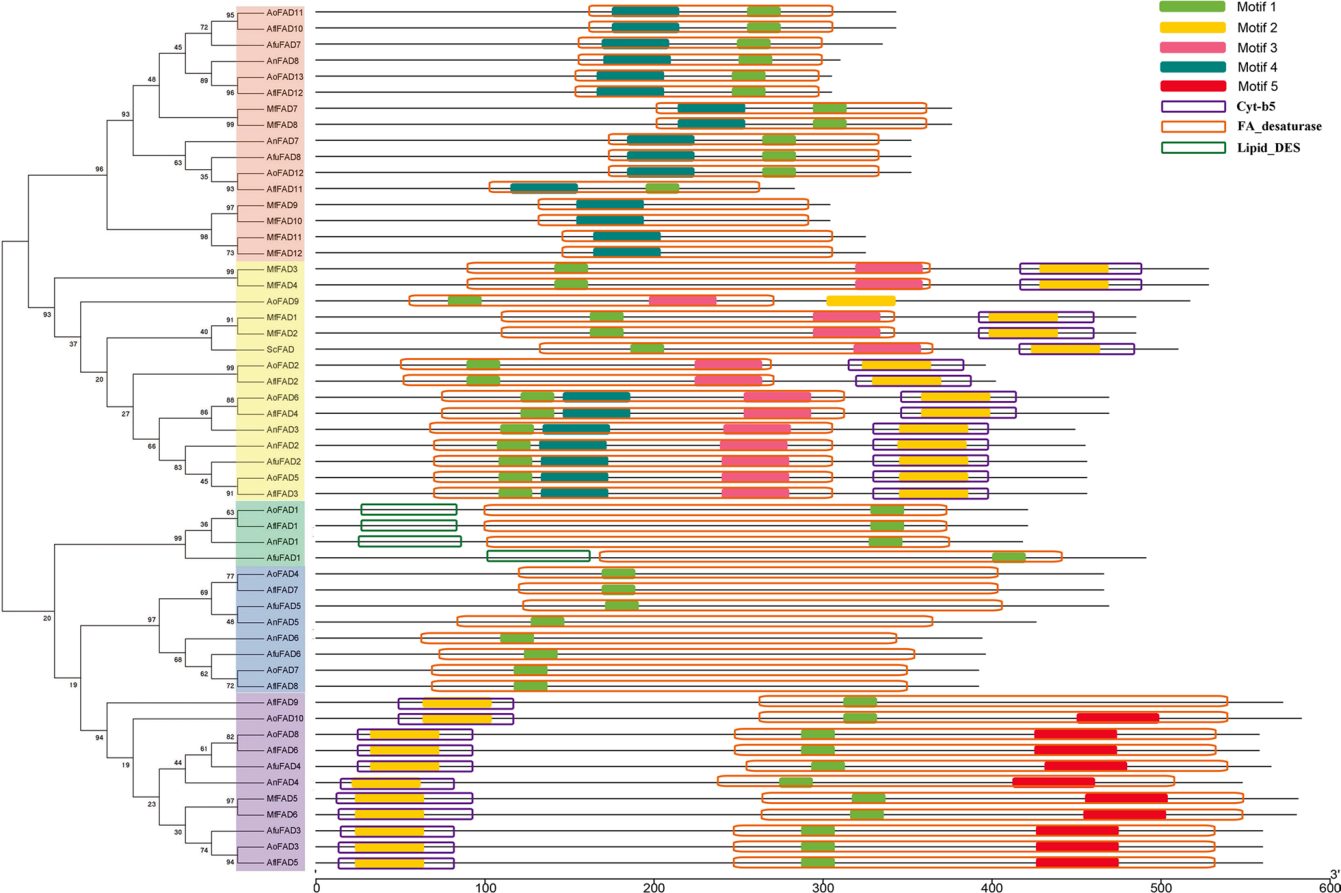
Motif analysis of fatty acid desaturases gene family of the six species. Motif compositions: protein sequences are indicated by thick gray lines, and the conserved motifs are represented by different colored boxes. The domains are displayed by different colored boxes without filling. The length (amino acids) of the protein and motif can be estimated using the scale bar at the bottom

Conserved motifs in the 54 fatty acid desaturases proteins were identified using the MEME program. A total of five conserved motifs were identified in the fatty acid desaturases proteins and their consensus sequence information was listed in Table 2. The logo of five conserved motifs identified in the fatty acid desaturases proteins were shown in Additional file 1: Figure S2. According to the phylogenetic tree and conserved motifs (Fig. 2), we could know that the same group of fatty acid desaturases genes had substantially consistent conserved motifs, which indicated there might be similar genetic functions. The fatty acid desaturases domains of most fatty acid desaturases gene members in group I-A contain motif 1 and motif 4, while most of group I-B fatty acid desaturases domains in group I-B all were consisted of motif 1, motif 2 and motif 3. The fatty acid desaturases domains were only composed of motif 1 in groups II-A and II-B-1. However, the fatty acid desaturases domains of subgroup II-B-2 were mainly consisted of motif 1, motif 2 and motif 5. The fatty acid desaturases domains consisting of different motifs in subgroup II-B-1 and subgroup II-B-2, suggested that functional differentiation might occur in the group II-B fatty acid desaturases genes. Besides, we found that motif 1, motif 3, motif 4 and motif 5 were contained in the fatty acid desaturases domains, while motif 2 was the part of Cyt-b5 domains, excepting the motif 2 in AoFAD9. The results revealed the conservation of motifs among various species.

**Table 2:** The information of motif found in MEME

Motif	Motif length	Motif sequence
1	38	HVITALVTLGEGYHNFHHEFPSDYRNAIEWYQYDPTKW
2	21	IGWWKRSHRVHHRYTBTPEDD
3	42	GRGJIIIGDVVHDVTAFIKFHPGGKKSIKHMVGKDATDEFNG
4	50	ISHMVTAPLHVQITLSHFAMSTADLGVNESFPQKMLRTTMDVDCPTWLDF
5	50	WTVMIHDGEYLANSPVVNGAACHTMHHLYFNYNYGQFTTLWDRLGGSYRK

### Gene structure analysis of the fatty acid desaturases genes

**Fig. 3:**
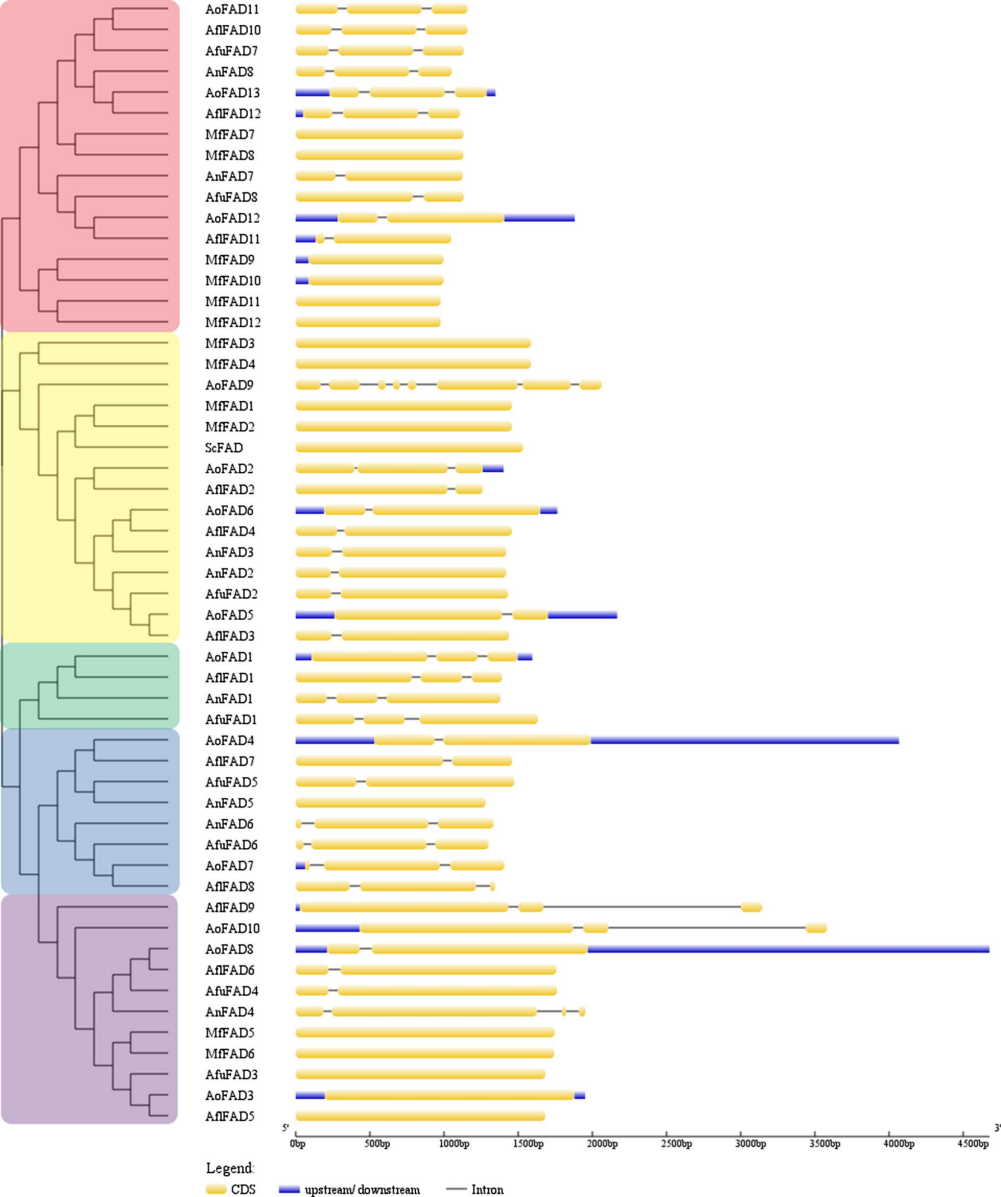
The gene structure analysis of fatty acid desaturases proteins based on their phylogentic relationships

In order to gain further insight into the structural diversity of fatty acid desaturases genes, coding sequences (CDSs) and corresponding genomic sequences were investigated through the six species. In the present study, a detailed illustration of the gene structures was shown in Fig. 3. The fatty acid desaturases genes of *A. oryzae* and *A. flavus*, clustered into a subbranch, had similar gene structures. The minor difference of the fatty acid desaturases gene structures between *A. oryzae* and *A. flavus* is that most of *AoFAD* contained upstream and downstream while only *AflFAD11* and *AflFAD9* contained an upstream sequence. And most of fatty acid desaturases genes in the two species possessed one or two introns except for *AoFAD9*, which had seven introns. The fatty acid desaturases genes of *S. cerevisiae* and *M. farinosa* were lacked of introns. Furthermore, the fatty acid desaturases genes of *M. farinosa*, which were clustered into the same branch, had same gene structures, further indicating the conservation of the fatty acid desaturases genes in *M. farinosa.*

### Expression of *AoFAD* genes during growth

**Fig. 4:**
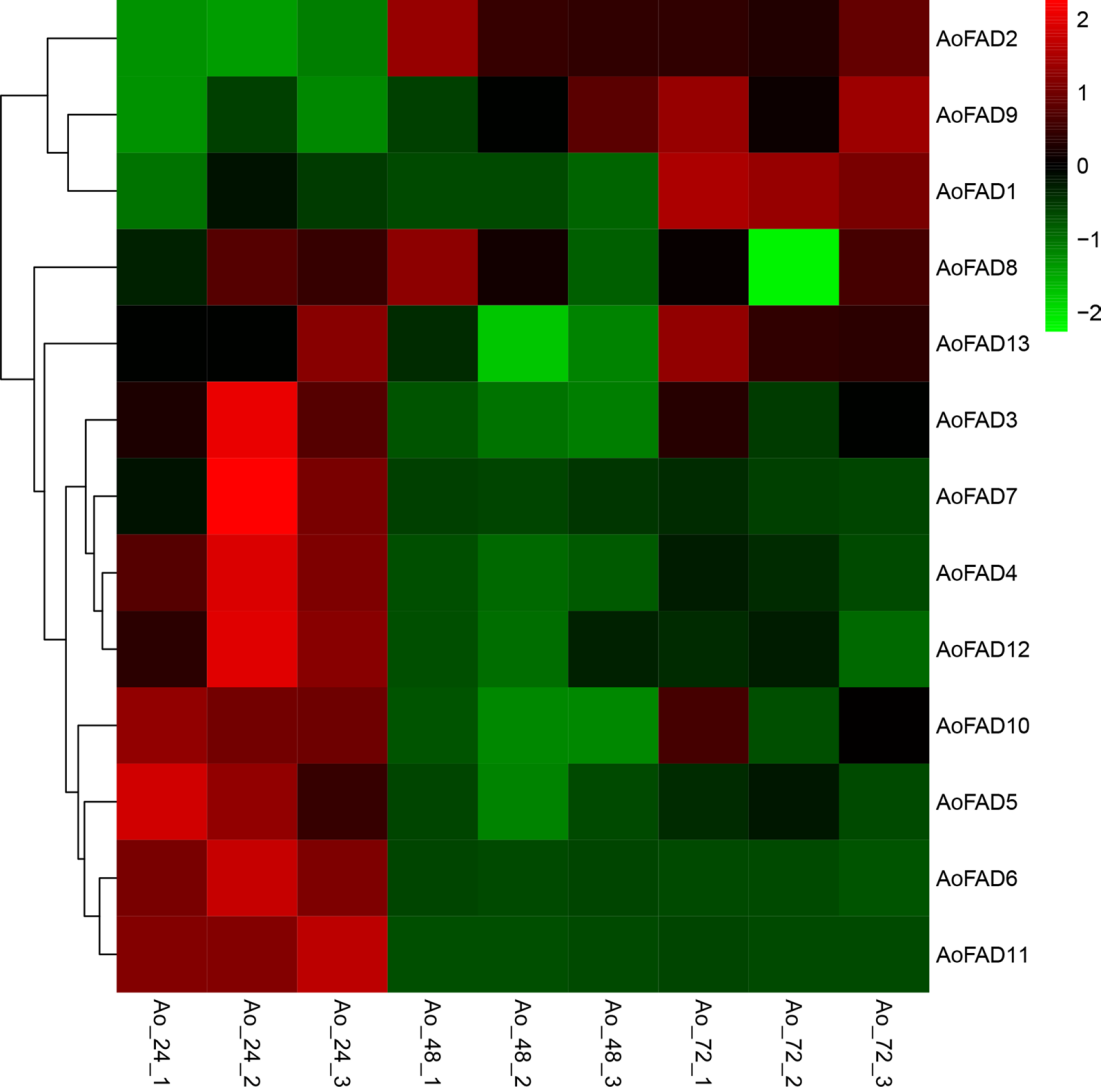
Expression of the *AoFAD* genes during growth. Ao_24_1, 2, 3 indicated the three biological reduplicates at 24 h (lag phase). Ao_48_1, 2, 3 indicated the three biological reduplicates at 48 h (logarithmic phase). Ao_72_1, 2, 3 indicated the three biological reduplicates at 72 h (stationary phase)

*Aspergillus oryzae* undergoes morphological differentiation across the different growth stages, which always accompany with the change of gene expression profile as well as metabolic pathways and influence the process productivity. To characterize the patterns of fatty acid desaturases gene expression during the growth stages of *A. oryzae*, samples at 24, 48 and 72 h (corresponding to the adaptive phase, logarithmic phase, and stationary phase), containing three biological replicates (i.e. Ao_24_1, 2, 3), were harvested. The expression patterns of the fatty acid desaturases genes at different growth periods in *A. oryzae* were shown in Fig. 4. Eight fatty acid desaturases genes, including *AoFAD3*, *AoFAD7*, *AoFAD4*, *AoFAD12*, *AoFAD10*, *AoFAD5*, *AoFAD6* and *AoFAD11*, showed the maximal expression in adaptive phase (Ao_24_1, 2, 3) and lower expression levels at logarithmic phase and stationary phase. *AoFAD1*, *AoFAD2* and *AoFAD9* were significantly up-regulated at 72 h, while the expression of *AoFAD8* and *AoFAD13* was obvious at 24 h and 72 h but not palpable at 48 h, indicating different roles of *AoFAD* with respect to the development of *A. oryzae*. To further confirm the expression profiles of *AoFAD*, six *AoFAD* genes were selected for qRT-PCR analysis. The results of qRT-PCR have strong consistency with those of transcriptome analysis (Additional file 1: Figure S1).

### Expression of *AoFAD* genes under salt stress

**Fig. 5:**
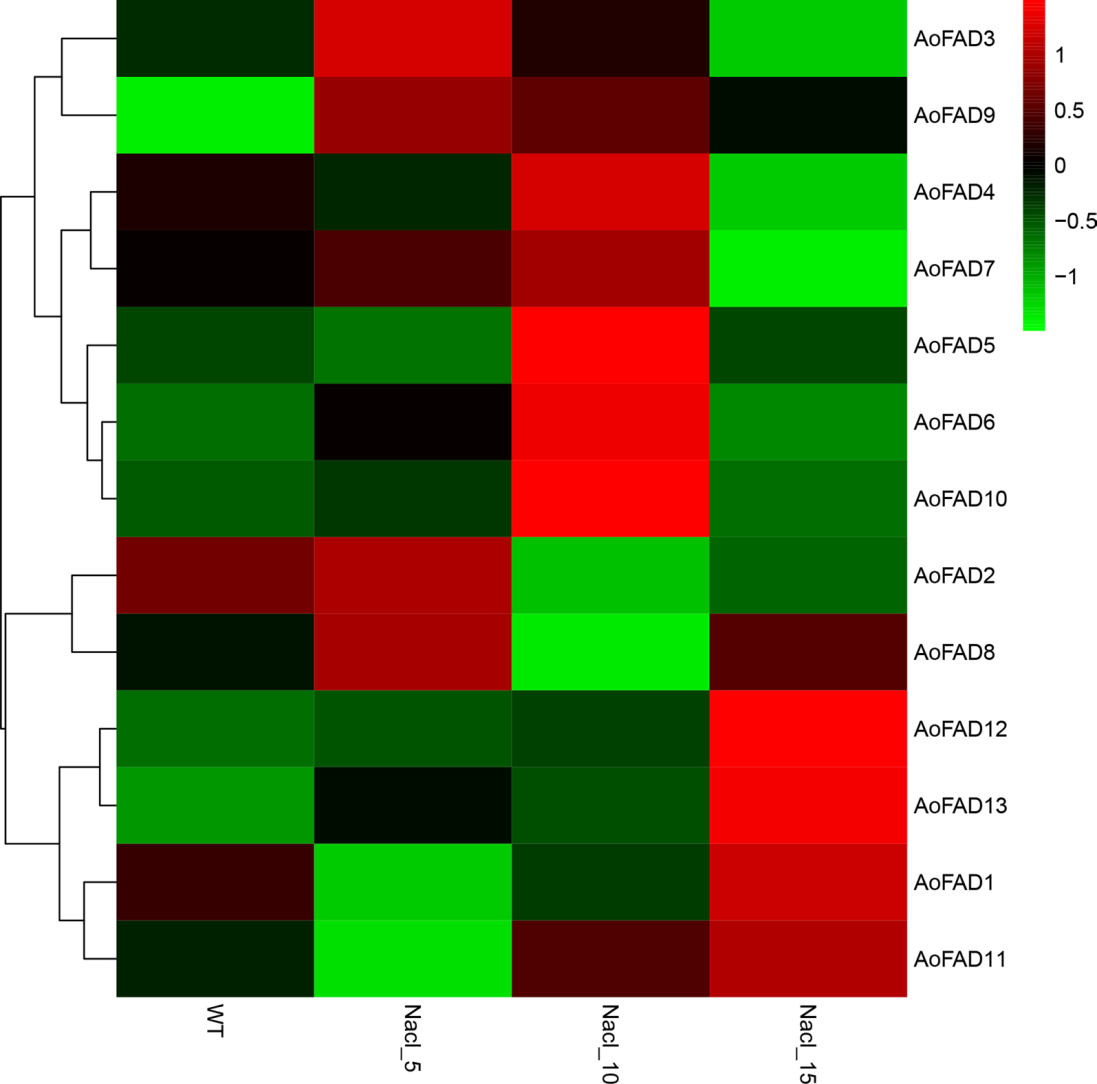
Expression of the *AoFAD* genes under salt stress. WT, NaCl_5, NaCl_10 and NaCl_15 indicated samples cultivated in PDA medium supplied with 0, 5, 10 and 15% NaCl, respectively

Unsaturated fatty acids play critical roles in the tolerance to various abiotic stresses, such as salt stress, cold stress, etc. (Sakamoto and Murata 2002).Therefore, gene expression patterns for all the fatty acid desaturases genes of *A. oryzae* were also observed under different levels of salt concentration. Results showed that the salt stress caused changes of the *AoFAD* expression patterns in the form of up-regulation (Fig. 5). Of these *AoFAD* genes, the expression of *AoFAD2*, *AoFAD3*, *AoFAD8* and *AoFAD9* reached the highest under 5% NaCl treatment. Four fatty acid desaturases genes in *A. oryzae,* including *AoFAD11*, *AoFAD1*, *AoFAD13* and *AoFAD12*, were highly expressed under 15% NaCl treatment. The expression level of *AoFAD4*, *AoFAD5*, *AoFAD6*, *AoFAD7* and *AoFAD10* was the highest under 10% NaCl treatment and was decreased under 15% NaCl treatment. The expression pattern of *AoFAD* genes under salt stress indicated that *AoFAD* are components of a complex transcriptional network regarding the salt stress and the mechanism of *AoFAD* genes involved in salt stress is complex and diversified. The coordination results of qRT-PCR were further confirmed the accuracy of analysis (Additional file 1: Figure S1).

## Discussion

Studies have indicated that fatty acid desaturases is expressed in plants, animals and fungi, and plays an important role in the synthesis of polyunsaturated fatty acid (Garba et al. 2016; Murphy and Piffanelli 1998). Fatty acid desaturases genes in fungi are responsible for multiple biological processes, from development and industrial production to adaption to the surrounding environment (Watanabe et al. 2010). Therefore, the study of the fatty acid desaturases genes is becoming a hot spot in the current biological research. So far, researchers have used DNA library, cDNA library and RT-PCR to clone fatty acid desaturases genes from plants, animals, bacteria, fungi and algae. For example, 29 and 21 fatty acid desaturases gene members were respectively identified from the fatty acid desaturases gene families of the soybean and *Arabidopsis thaliana* (Chi et al. 2011). Besides, there are some reports on the cloning and expression of fatty acid desaturases in microbes. For example, the sole fatty acid desaturases gene in *Bacillus subtilis*, named *des*, encoding Δ5-desaturase, was cloned by Ma and Liu (Ma and Liu 2010). The expression of three fatty acid desaturases genes in the *Cytosolic cyanobacteria*, named *desA, desB and desD*, was up-regulated under low temperature (Los et al. 1997). *Aspergillus* species has been intensively used for the production of traditional fermented foods and secondary metabolite, such as fumagillin. Therefore, the synthesis of unsaturated fatty acid appears to be essential and vital for the *Aspergillus* species to adapt to some complex environments and regulate the growth as well as development. However, systematic investigations of the fatty acid desaturases gene family at the whole-genome level was absent in *Aspergillus* species. Therefore, a comprehensive survey of fatty acid desaturases gene family in *Aspergillus* species was undertaken.

The results in this study showed that all of the 54 fatty acid desaturases genes in the six fungi species contained FA_desaturase domain and most of the fatty acid desaturases genes had Cyt-b5 domain as well. These findings echo previous studies which studied the regulation of fatty acid synthesis pathways in the *Caenorhabditis elegans*. He et al. (2017b) reported that, in the desaturation of fatty acids, electrons are transferred to cytochrome b5 through cytochrome b5 reductase, which activates desaturase, and introduces unsaturated bonds to the unsaturated fatty acids at the specific location of the carbon chain of fatty acids (He et al. 2017b). When the fatty acid desaturases genes were absent of the Cyt-b5 domain, there need to be additional cytochrome b5 reductase to transfer electrons. From their result, we could infer that only FA_desaturase domain was specifically required for the activity of some fatty acid desaturases while some fatty acid desaturases were activated by FA_desaturase and Cyt-b5 domain. The other researches showed that some of the fatty acid desaturases were activated by FA_desaturase and DUF3474 domain (He et al. 2017b). In our study, the fatty acid desaturases genes without the Cyt-b5 domain existed in *Aspergillus* species as well, which may be need additional cytochrome b5 reductase to transfer electrons. In addition, we found a fatty acid desaturases gene in *A. nidulans* (*AnFAD5*) which depends on FA_desaturase and DUF3474 domain to activate. However, the functions of some domains identified in the fatty acid desaturases genes of *Aspergillus* species, such as DUF953, were not clear.

The relationship of *A. oryzae* and *A. flavus* is a controversial issue which has long been plagued with researchers. A very strong phylogenetic connection between *A. oryzae* and *A. flavus* has been clearly demonstrated by molecular methods, including isozyme analyses, DNA/DNA hybridization studies and DNA sequencing (Chang et al. 2006; Geiser et al. 2000). Furthermore, the morphological characteristics and genomes of the two *Aspergillus* species were similar, which was thus difficult to distinguish. In this study, there were 13 fatty acid desaturases genes identified in *A. oryzae*, whereas 12 fatty acid desaturases genes were identified in *A. flavus*. From the phylogenetic tree, the 12 fatty acid desaturases genes from *A. flavus* were all clustered with *A. oryzae* into a sub-branch, which supported a close relationship between the *A. oryzae* and *A. flavus*. Additionally, *AoFAD9* was grouped closely with *MfFAD1*, *2*, *3* and *4*, which could be considered as a method to distinguish *A. oryzae* and *A. flavus*.

In this study, analysis of the *AoFAD* expression profiles showed that the different concentration of salt stress caused changes of the *AoFAD* expression patterns in the form of up-regulation. The results revealed that *AoFAD* genes were assumed to be associated with salt stress, which has been mentioned in the previous studies (He et al. 2017a). The potential mechanism was that the increase of unsaturated fatty acids is beneficial to maintain membranes in an appropriate fluid state, which counteracts the fluidizing effect of salt stress. In fact, there are many studies which convey that the fatty acid desaturases genes have a closed relation with the salt stress in many species. For example, in the *Arabidopsis thaliana*, the *FAD2* and *FAD6* are essential for improving the early growth and salt tolerance of the seedlings while the antisense expression of *FAD7* gene reduces plant tolerance to salt stress (Zhang et al. 2009, 2012). In addition, the overexpression of *LeFAD3* gene can enhance the salt tolerance of early growth of the tomato seedlings (Wang et al. 2014). Our results, taken together with these earlier studies, imply that the fatty acid desaturases genes have an effect on salt stress.

## Additional file

**Additional file 1.** Additional table and figures.

## Abbreviations

FA: fatty acid
*A. oryzae*: *Aspergillus oryzae*
*S. cerevisiae*: *Saccharomyces cerevisiae*
*M. farinosa*: *Millerozyma farinosa*
*A. flavus*: *Aspergillus flavus*
*A. fumigatus*: *Aspergillus fumigatus*
*A. nidulans*: *Aspergillus nidulan*
FPKM: fragments per kilobase of transcript per million mapped reads
PUFA: polyunsaturated fatty acids
GLA: gamma linolenic acid
ARA: arachidonic acid
