# Supplementary material for: Genome-wide identification of the fatty acid desaturases gene family in four Aspergillus species and their expression profile in Aspergillus oryzae
Source: AMB Express. 2018 Oct 15;8:169. doi: 10.1186/s13568-018-0697-x (PMC6188973; doi:10.1186/s13568-018-0697-x)
Supplement: Supplementary file 1 — Additional file 1. Additional table and figures. [file 13568_2018_697_MOESM1_ESM.pdf]

Genome-wide identification of the fatty acid desaturases gene family in four Aspergillus species and their expression profile in Aspergillus oryzae

Wen Tang [a,#] , Changsheng Ouyang [b,c,#] , Lanlan Liu [a] , Haoran Li [a] , Chuanhui Zeng [a] , Jie Wang [a] , Lijun Fu [a] , Bin Zeng [a,*] and Bin He [a,*]

[a] Jiangxi Key Laboratory of Bioprocess Engineering and Co-Innovation Center for In-vitro Diagnostic Reagents and Devices of Jiangxi Province, College of Life Sciences, Jiangxi Science & Technology Normal University, Nanchang 330013, China

[b] Nanchang University, Nanchang 330013, China

[c] Jiangxi provincial people's hospital, Nanchang 330006,China

[#] These authors contributed equally to this work

* Corresponding Author

Email: Zengtx001@aliyun.com; hebin.li@foxmail.com. Tel/Fax: 860791-88539361.

Additional file 1: Table S1. qRT-PCR primers used in this study

Primer	Sequence (5′-3′)	Purpose
AoFAD1-F	CTCTGTTCCGTGATCGACTTT	qRT-PCR of AoFAD1
AoFAD1-R	CAGCCCTTCTTCTACCTCATTC	qRT-PCR of AoFAD1
AoFAD2-F	CCGAAGGCAGCTAAGAAGTAATA	qRT-PCR of AoFAD2
AoFAD2-R	GTCAGCCTCGGAATGAAAGA	qRT-PCR of AoFAD2
AoFAD3-F	CCTGACAGAGCGTAATAGGAATAG	qRT-PCR of AoFAD3
AoFAD3-R	CTGCAATCCGAAGGACTCTAC	qRT-PCR of AoFAD3
AoFAD4-F	GACTCGATCGACAGGCAATTAG	qRT-PCR of AoFAD4
AoFAD4-R	GCATGGACGACGAGAAGTTAG	qRT-PCR of AoFAD4
AoFAD5-F	CCTGCTGGACGAAGAAGATACG	qRT-PCR of AoFAD5
AoFAD5-R	TGGGTCTGTTTGTGCCTATG	qRT-PCR of AoFAD5
AoFAD6-F	CTCGCCTACGATCTCAAACAA	qRT-PCR of AoFAD6
AoFAD6-R	GAACTCGTCCCAGTCCATTAC	qRT-PCR of AoFAD6
AoFAD7-F	AGAAGAACGTGCGGATTGAG	qRT-PCR of AoFAD7
AoFAD7-R	GTGGCATTCAGGAACAGATAGA	qRT-PCR of AoFAD7
AoFAD8-F	GGCCAAGTCTTCTTCTGGTA	qRT-PCR of AoFAD8
AoFAD8-R	CGGTAACCATGTGCGAGATTA	qRT-PCR of AoFAD8

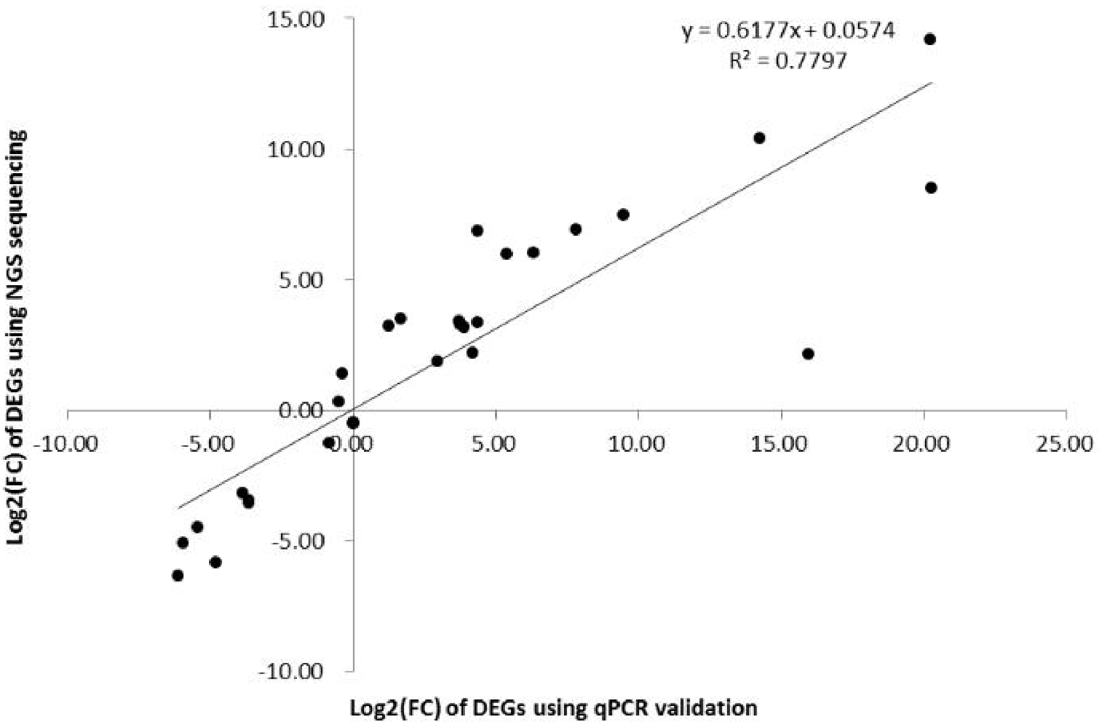

Additional file 1: Figure S1. Correlation analysis of qRT-PCR and transcriptome results.

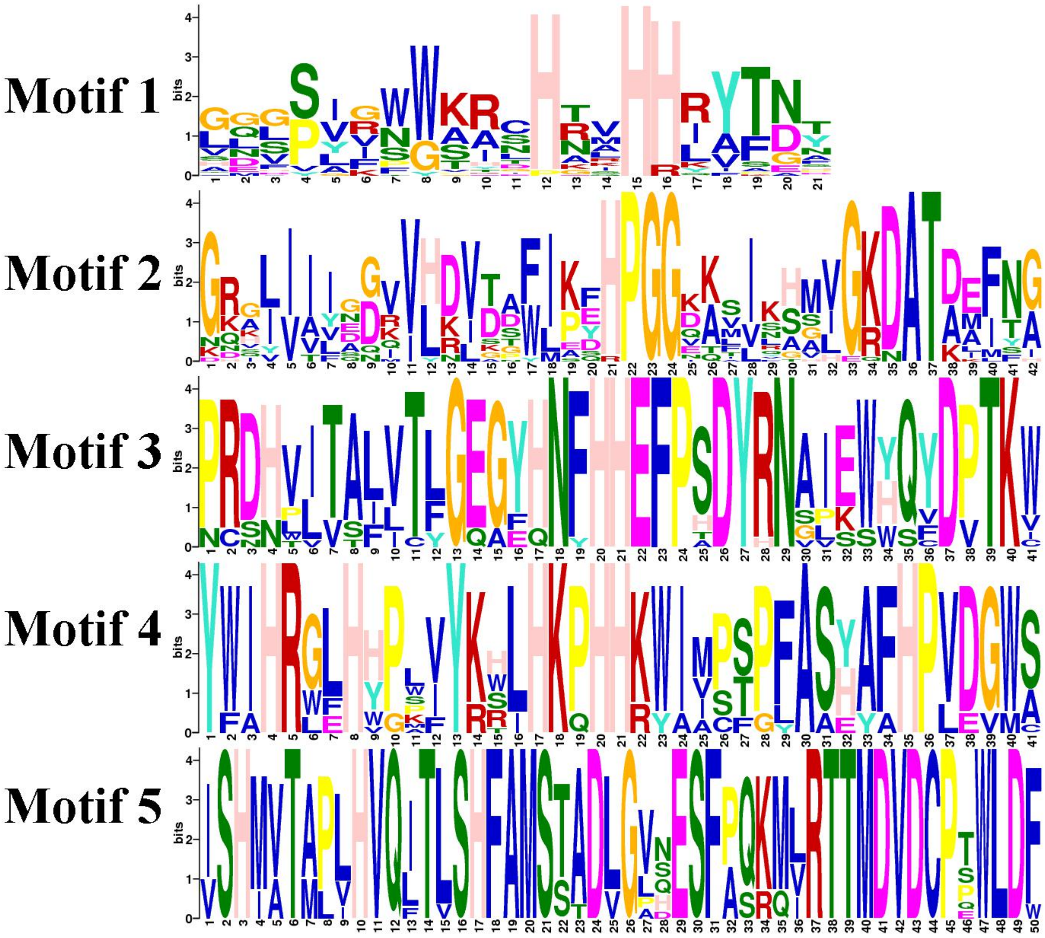

Additional file 1: Figure S2. Sequence logos of conserved motifs.
